# Photo-reactive charge trapping memory based on lanthanide complex

**DOI:** 10.1038/srep14998

**Published:** 2015-10-09

**Authors:** Jiaqing Zhuang, Wai-Sum Lo, Li Zhou, Qi-Jun Sun, Chi-Fai Chan, Ye Zhou, Su-Ting Han, Yan Yan, Wing-Tak Wong, Ka-Leung Wong, V. A. L. Roy

**Affiliations:** 1 Department of Physics and Materials Science, City University of Hong Kong, Kowloon Tong, Hong Kong SAR; 2 Department of Applied Biological and Chemical Technology, The Hong Kong Polytechnic University, Hung Hum, Hong Kong SAR; 3 Department of Chemistry, Hong Kong Baptist University, Kowloon Tong, Hong Kong SAR

## Abstract

Traditional utilization of photo-induced excitons is popularly but restricted in the fields of photovoltaic devices as well as photodetectors, and efforts on broadening its function have always been attempted. However, rare reports are available on organic field effect transistor (OFET) memory employing photo-induced charges. Here, we demonstrate an OFET memory containing a novel organic lanthanide complex Eu(tta)_3_ppta (Eu(tta)_3_ = Europium(III) thenoyltrifluoroacetonate, ppta = 2-phenyl-4,6-bis(pyrazol-1-yl)-1,3,5-triazine), in which the photo-induced charges can be successfully trapped and detrapped. The luminescent complex emits intense red emission upon ultraviolet (UV) light excitation and serves as a trapping element of holes injected from the pentacene semiconductor layer. Memory window can be significantly enlarged by light-assisted programming and erasing procedures, during which the photo-induced excitons in the semiconductor layer are separated by voltage bias. The enhancement of memory window is attributed to the increasing number of photo-induced excitons by the UV light. The charges are stored in this luminescent complex for at least 10^4^ s after withdrawing voltage bias. The present study on photo-assisted novel memory may motivate the research on a new type of light tunable charge trapping photo-reactive memory devices.

Organic field-effect transistors (OFETs) have received considerable attention in the past two decades owing to their advantages of low cost, light weight, flexibility and simpler manufacturing process compared with the conventional silicon based electronics^1^,^2^,^3^,^4^,^5^,^6^. Owing to these advantages, diverse OFET based devices such as sensors, phototransistors, light emitting transistors, and memories have been widely studied^7^,^8^,^9^,^10^. Among them, OFET based memory is the category of devices having two or more electric states so that information can be stored on the basis of different electric responses^11^,^12^,^13^. In a memory device, the most important characteristics are memory window, operation speed, cycling endurance as well as data retention capability^14^,^15^. Among these criteria, the memory window, defined as the shift of threshold voltages when a device is undergone programing and erasing processes, is extremely important and represents the ability of data storage^16^,^17^,^18^. Tremendous efforts have been made to enlarge the memory window by employing novel materials, optimizing available candidates or designing novel device architectures and so on^18^,^19^,^20^,^21^,^22^. Although impressive achievements have been obtained, there are always limitations on materials selection and device architecture. Therefore, it is necessary to explore new materials, device architecture.

Recently, the reports on transistor devices with light controllable threshold voltage shifts have attracted a lot of attention as these reports may provide a method that could be utilized for the optimization of memory window in memory devices^23^,^24^,^25^. The idea mainly focuses on the application of photo-induced charge carriers. Traditionally, these carriers are used as tools of detecting photons in FET-based photodetectors, which have electrical responses when they are irradiated by light with specific wavelength. The demonstrations of controlling threshold voltage with the assistance of light proved the possibility of making full use of these photo-generated charges in memory device^26^,^27^,^28^. In that case, the light can be regarded as the fourth terminal compared with the conventional FET devices with source, drain and gate electrodes^29^,^30^,^31^. Furthermore, multi-level memory could be attained in photo-reactive memory by a combination of bias voltage and photo-irradiation^10^,^32^,^33^. Up to now, several sorts of memory devices containing photo-reactive component, either the semiconductor and dielectric, or the modification layer, have been fabricated and the performance has been improved with the assistance of light^28^,^34^.

Here, we demonstrate an ultraviolet (UV) light-assisted programmable and erasable pentacene device with novel photo-reactive organic charge trapping complex, Eu(tta)_3_ppta. The organic luminescent lanthanide complex was chosen due to the compatibility with OFETs and high luminescent quantum efficiency. The complex serves as the charge trapping layer and the memory window can be increased apparently with the assistance of UV light. The organic complex, which has an intense response upon UV light excitation, has first ever been applied as the charge trapping element in an organic memory. The memory window and data retention capability have been studied. The study indicates that the photo-reactive complex Eu(tta)_3_ppta can be a good candidate of charge trapping element and the approach of utilizing light has great potential in optimizing performance of the OFET memories.

## Results

### Charge trapping layer and semiconductor properties

**Figure 1:**
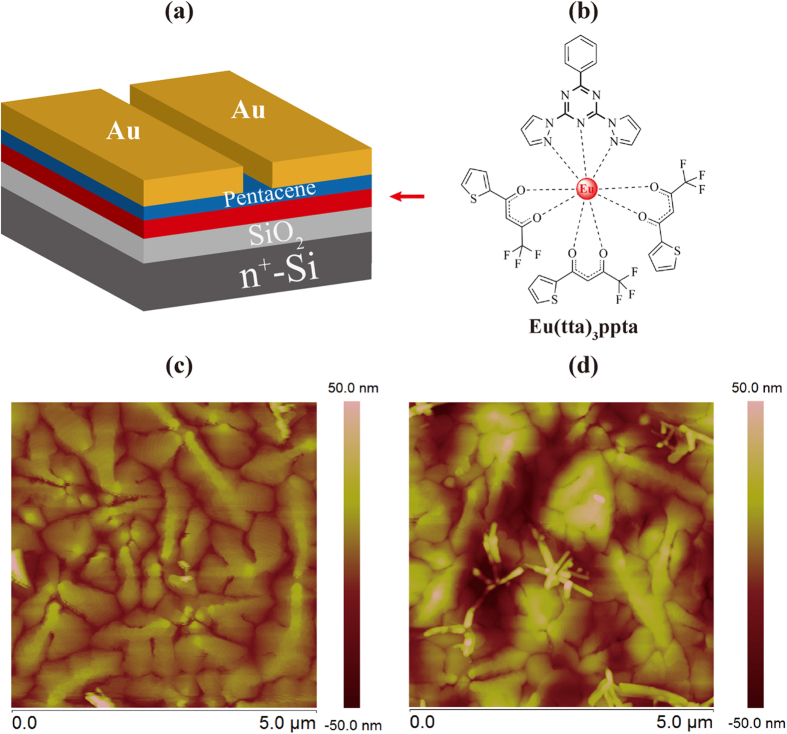
(**a**) Schematic diagram of the photo-reactive memory device and (**b**) the molecular structure of charge trapping photo-reactive Eu(tta)_3_ppta complex. AFM images of pentacene grown on (**c**) bare SiO_2_ and (**d**) Eu(tta)_3_ppta film.

The bottom-gate top-contact structure containing Eu(tta)_3_ppta charge trapping layer is shown in Fig. 1a. Figure 1b presents the molecular structure of the Eu(tta)_3_ppta charge trapping layer in this study. The structure of prepared Eu(tta)_3_ppta was confirmed by ^1^H NMR spectroscopic data given in Fig. S1 (Supplementary Information). Furthermore, the Eu(tta)_3_ppta can be excited by UV light and emit red emission due to the *f*-*f* transaction of rare earth Eu^3+^ ion^35^. Figure 1c,d show the AFM images of pentacene film grown on a bare SiO_2_ and Eu(tta)_3_ppta film, respectively. Obvious difference in morphology of pentacene can be observed due to the rough surface of Eu(tta)_3_ppta complex. (see Fig. S2 in the Supplementary Information). In general, a rough surface of underlying layer would generate an inferior conduction channel when voltage bias is applied. However, in the present study, a direct contact between the rough complex surface and pentacene would benefit the charge injection efficiency from pentacene to the complex. Therefore, we proposed that the abundant interface between the Eu(tta)_3_ppta and pentacene molecules could efficiently increase the contact area so that the hole trapping efficiency is enhanced. In addition, the complex (Eu(tta)_3_ppta) consists of donor-acceptor blocks and a large conjugated system in the ligand (ppta) and the Eu(tta)_3_, respectively, providing charge trapping sites for the designed memory^36^,^37^,^38^. It is possible to optimize the growth of pentacene through surface treatment on Eu(tta)_3_ppta film^39^. However, the direct interaction between Eu(tta)_3_ppta complex and pentacene is critical for this study as the modification layer will increase the barrier for charge transport.

**Figure 2:**
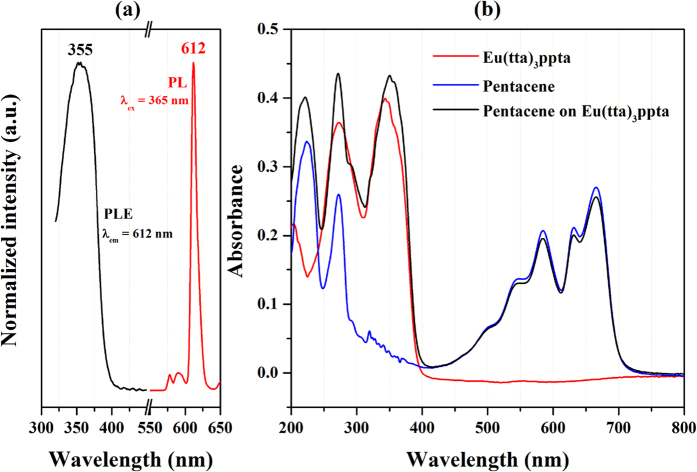
(**a**) The PL and PLE spectra of Eu(tta)_3_ppta film. (**b**) The UV-vis absorbance spectra of pure Eu(tta)_3_ppta film, pure pentacene, and pentacene grown on Eu(tta)_3_ppta film.

To explore the photoluminescent properties of this novel compound, we characterized the photoluminescence emission (PL) and excitation (PLE) spectra of Eu(tta)_3_ppta film spin-coated on quartz glass. As shown in the Fig. 2a, the complex Eu(tta)_3_ppta can be excited upon a broad band UV light from 325 to 400 nm and emits red emission^35^. Furthermore, the peaks with maximum intensities for PLE and PL spectra are 355 nm and 612 nm, showing a red and blue shift compared with the results of Eu(tta)_3_ nanocrsystalline, respectively. In principle, the luminescence of Eu^3+^ originates from the *f*-*f* transition with sharp line emission and is less sensitive to the surrounding environment^35^,^40^,^41^. Therefore, the observed shift in the emission is acceptable and the cause of these phenomena is due to the introduction of ligand ppta. Furthermore, the emission spectrum of Eu^3+^ ion obviously presented the characteristic fluorescence of Eu^3+^ ion due to the energy transfer between the tta and Eu^3+^ ion^35^. It is well known that active charges will be generated in semiconductor material if the photon energy of incident light is equal to or larger than its band gap. It is notable that, in this study, the emitting light with wavelength of 612 nm has a photon energy of ~2.0 eV according to the general expression E_hu_(eV) = 1240/[λ(nm)]^42^. This red emission can also excite the semiconductor pentacene, which has an energy gap of ~1.9 eV between its lowest unoccupied molecular orbital (LUMO) and highest occupied molecular orbital (HUMO) so that more photo-induced charges can be utilized^23^,^43^,^44^,^45^.

Figure 2b shows the UV-vis absorbance spectra of Eu(tta)_3_ppta film, pristine deposited pentacene and pentacene on Eu(tta)_3_ppta film. The Eu(tta)_3_ppta shows strong absorption in the UV light region, especially from 300 to 400 nm. It is quite obvious that the absorption of pentacene on Eu(tta)_3_ppta increased significantly compared to that of pristine pentacene in the wavelength region mentioned above. For the UV light assisted measurements, we use a commercial UV LED as the light source and the spectral overlap of UV LED light source, the excitation and absorption of pentacene with Eu(tta)_3_ppta is given in Fig. S3 (Supplementary Information). The improvement of photon absorption in this region provides an opportunity to employ light as a control terminal other than the conventional source, drain and gate electrodes in this device architecture.

### Electronic properties of charge trapping memory

**Figure 3:**
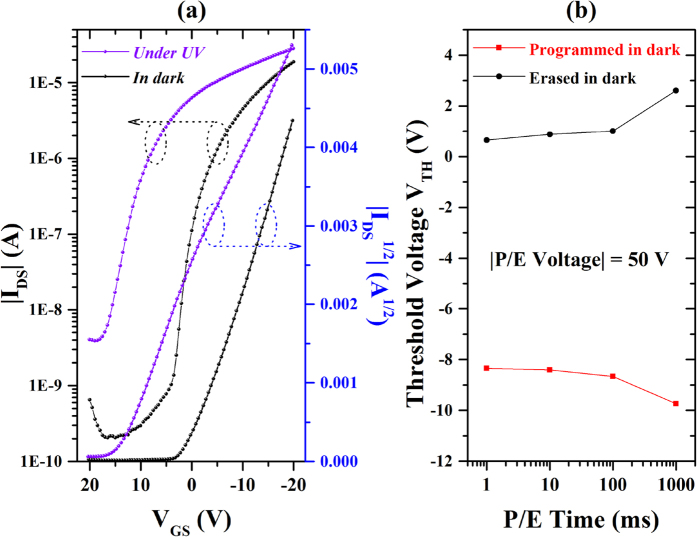
(**a**) Transfer characteristics of device with Eu(tta)_3_ppta film measured in dark and under UV irradiation with a power density ~200 μW/cm^2^. (**b**) The relationship of threshold voltage V_TH_ and the programming/erasing duration in dark environment.

To characterize the memory effect of the complex, we fabricated memories on commonly used Si substrate with 100 nm thick SiO_2_ dielectric. In general, low operating voltage could be realized by using thinner atomic layer deposited (ALD) Al_2_O_3_ or HfO_2_ to reduce the power consumption. However, as a proof of concept, here we focus on the novelty on the memory effect of the lanthanide complex. Figure 3a shows the typical transfer characteristics of a device using Eu(tta)_3_ppta film as charge trapping layer. The pentacene memory exhibits typical p-type field-effect behavior, operated under a negative bias voltage with drain-source voltage of −20 V (V_DS_ = −20 V). In the absence of UV light irradiation, the saturation mobility, current on-off ratio of this device are determined to be 0.16 cm^2^·V^−1^·s^−1^ and 9.4 × 10^4^, while for a control device with bare SiO_2_, the values are 0.31 cm^2^·V^−1^·s^−1^ and 5.9 × 10^5^, as given in Fig. S4 (Supplementary Information). The decrease in mobility and on/off ratio is due to the inferior surface quality of Eu(tta)_3_ppta for pentacene growth, which can be seen from the AFM image given in Fig. 1d. However, these results are comparable or even better than the reported results, in which light is also employed as a control terminal^23^,^26^. It is interesting that the transfer curve has a large shift under UV light irradiation with a power density of ~200 μW/cm^2^, as shown in Fig. 3a. The drain current increases significantly due to the carriers generated in pentacene layer by UV light excitation compared with the control device, as given in Fig. S4 (Supplementary Information). Furthermore, the threshold voltage shifts from −1.21 V to 15.12 V, while it shifts from 0.07 V to 2.55 V for control device as shown in Fig. S5 (Supplementary Information). The reason of this phenomenon is proposed to be the excitation by the UV light and red emission of the luminescent complex. To investigate the programming and erasing speeds of the device in dark, negative and positive bias voltage (±50 V) were applied for various durations from 1 ms to 1000 ms. The result of threshold voltage shift versus program/erase durations is shown in Fig. 3b, the memory window increases from ~9 V to ~12 V with longer bias time. Here, a memory window of 10 V, which is 20% of the voltage bias, is obtained within 100 ms, presenting a faster operation speed than the memories using oxide or nanoparticles as charge trapping layer^11^,^18^. In this work, the duration was fixed to 1 s due to the precision of UV light switching setup.

**Figure 4:**
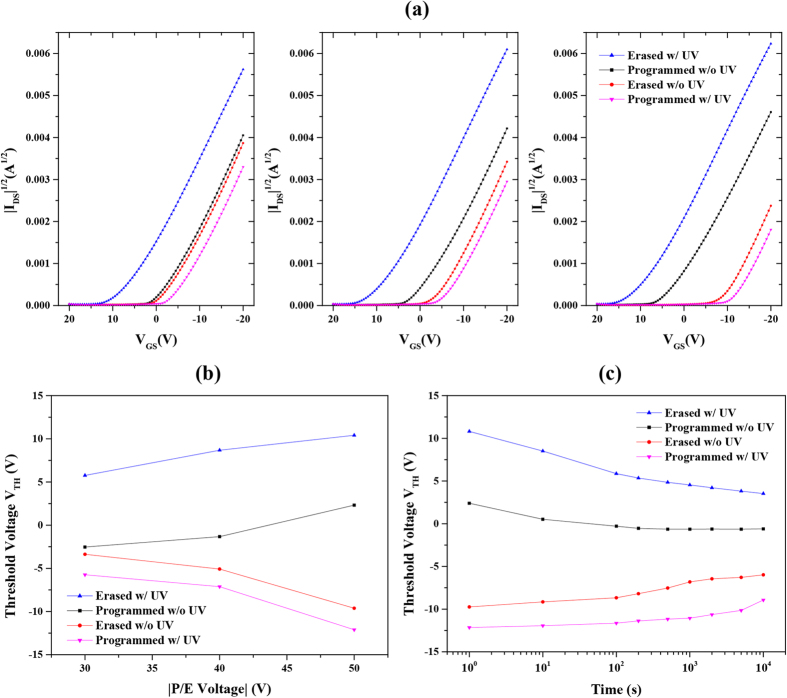
(**a**) The transfer characteristics of device with Eu(tta)_3_ppta film at programmed (by applying negative bias voltage), erased states (by applying positive bias voltage) after operating at |30| V, |40| V and |50| V for 1 s with and without assistance of UV light. (**b**) The relationship of threshold voltage V_TH_ and the programming/erasing voltages of devices operated with and without assistance of UV light. (**c**) Data retention as a function of elapsed time of device at programmed and erased states operated with and without assistance of UV light.

In order to demonstrate the utilization of photo-induced charges, we compared the device performance when operated with or without UV light irradiation. Figure 4a shows the transfer curves of device with Eu(tta)_3_ppta film programmed and erased at various bias voltages for 1 s with and without assistance of UV light. It is notable that the threshold voltage shifts are observed by applying positive and negative bias voltage for 1 s either in dark environment or under UV light irradiation, as shown in Fig. 4b. Furthermore, we can conclude that the complex Eu(tta)_3_ppta serves as a hole trapping element in this memory as the threshold voltage shifts to negative direction after applying a negative bias voltage. Additionally, the memory windows are 0.8 V, 3.8 V and 11.9 V when the device is operated in dark at ±30 V, ±40 V and ±50 V, respectively. With the assistance of UV light irradiation with the voltage bias, the memory windows are enlarged to 11.5 V, 15.8 V and 22.5 V, respectively. An impressive memory window (e.g. 45% of the operated bias voltage of ±50 V) is obtained by the proposed UV light-assisted operation compared to that of floating gate memories, showing its great potential in memory window enhancement^11^,^18^. Moreover, the current ratios (V_GS_ = V_DS_ = −20 V) increased from 1.1, 1.5 and 3.8 in dark to 2.9, 4.3 and 11.9 under UV light after erasing and programing operations at ±30 V, ±40 V and ±50 V, respectively. Therefore, the UV light employed in this study can be regarded as a fourth control terminal that controls the channel transport and we believe the approach of utilizing light is noteworthy in motivating the study on photo-reactive memory devices. To investigate the performance of charge retention capability, the threshold voltage shift, has been monitored to estimate the charge loss after the application of ±50 V for 1 s with or without the irradiation of UV light. As shown in Fig. 4c, the percentages of charges remained in the complex compared with its initial states increased from 44.3% to 54.3% after 10^4^ s, which results from the generation of more charges using the assistance of UV light. In addition, the obtained results show comparable charge retention capability with other pentacene memories, indicating its suitability as a charge trapping material. The inevitable charge loss is ascribed to the results of charge leakage from the shallow traps by the repulsion of F and O atoms due to its large electron affinity at the interfaces as well as charge de-trapping by photo-excitation of light in the surrounding environment^46^,^47^,^48^. The retention capability could be improved by inserting a tunnelling layer in the current device architecture. In addition, as the incorporated complex is sensitive to the photons, which makes the device unstable under light sources containing UV and the stored charges could be excited inevitably. Therefore, surface passivation is necessary to improve the charge retention capability.

### Working mechanism under UV light irradiation

**Figure 5:**
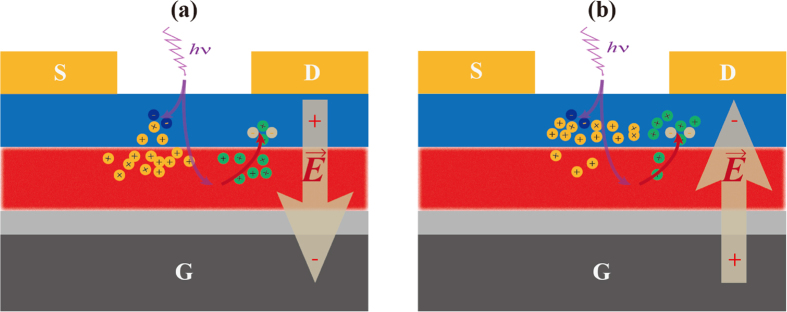
Proposed principal diagram of device with Eu(tta)_3_ppta film operated at (a) programming and (b) erasing modes with assistance of UV light. The arrows in violet and red color represent partial UV light and red emission by Eu(tta)_3_ppta film.

To explain the principle of enhancement on memory window, we proposed a working mechanism as shown in Fig. 5. The incident UV light can be divided into two parts, as shown in Fig. 5. Apparently, some portion of UV light will directly excite the pentacene semiconductor followed by the generation of electrons and holes^27^,^28^. The rest of incident light excites the Eu(tta)_3_ppta film and emits red light. A strong excitation of pentacene is caused by the red emission from the Eu(tta)_3_ppta film, as a direct contact between the complex and pentacene is obvious in this system. Moreover, the photon energy of red emission from the complex is larger than the gap between LUMO and HOMO of pentacene. The holes and electrons either existed intrinsically in pentacene or generated by the UV and red light are separated by the application of voltage bias. Therefore, larger memory windows are obtained with the assistance of UV light irradiation as there are more photo-induced charges in both programming and erasing processes.

## Discussion

In summary, we have demonstrated a novel lanthanide complex as charge trapping layer in the OFET based memory device for the first time. The solution processed organic complex, Eu(tta)_3_ppta, emits intense red emission upon UV light excitation. The memory window of the device with photo-reactive Eu(tta)_3_ppta charge trapping layer is enhanced from 11.9 V to 22.5 V with the assistance of UV light. In addition, the charge retention capability improved from 44.3% to 54.3% of its initial programmed and erased states with assistance of UV light. The current study indicates that novel lanthanide complexes are good candidates as charge trapping layer for OFET memory, and the application of light as a fourth terminal to control over the channel conductance in the memory is a potential tool to optimize the performance of OFET based memory with photo-reactive element.

## Methods

### Synthesis of complex Eu(tta)_3_ppta

First, phenylmagnesium bromide was dropped into a tetrahydrofuran (THF) solution of cyanuric chloride at 4 °C and stirred at 4 °C for 4 h. Second, the reaction mixture was quenched with saturated ammonium chloride and extracted with diethyl ether and water. Third, the organic portion was evaporated and dichlorophenyl-1,3,5-triazine was obtained by column chromatography using hexane and dichloromethane (DCM) with a volume ratio of 3 to 1. Then, 3.5 equivalents of 3-methylpyrazole were deprotonated by refluxing with potassium metal in THF for 3 hours. Upon cooling to room temperature, a THF solution of dichlorophenyltriazine was added at room temperature and stirred for a further hour before refluxing overnight. The solvent mixture was subjected to extraction with DCM and water. Finally, the organic portion was evaporated and the ligand 2-phenyl-4,6-bis(pyrazol-1-yl)-1,3,5-triazine (ppta) was obtained by column chromatography using ethyl acetate as the eluent^49^,^50^. Commercial Europium(III) thenoyltrifluoroacetonate hydrate (Eu(tta)_3_·3H_2_O) and the as-prepared ligand (ppta) was mixed in a 1:1 ratio in a solution of methanol and stirred at 50 °C overnight. The solvent was subsequently evaporated and the residue was re-dissolved in minimal amount of diethyl ether. The product Eu(tta)_3_ppta powder was obtained as a white/very pale yellow solid by precipitating with n-hexanes.

### Device fabrication

The memory device with bottom-gate top-contact architecture was constructed due to the simplicity of the fabrication processes. In a typical procedure, heavily doped *n*-type silicon wafer with 100 nm thick oxide layer was cleaned ultrasonically in deionized water, acetone and ethanol each for 15 min, and dried by N_2_ gas and kept in oven at 120 °C for 2 h. The as-prepared photo-reactive Eu(tta)_3_ppta powder was dissolved in methanol solution with a fixed concentration of 10 mg/ml and filtered through 0.2 μm PTFE filter. The light yellow solution was then spin-coated at 4000 rpm for 40 s and heated on hotplate at 120 °C for 10 min in nitrogen atmosphere. After the Eu(tta)_3_ppta film formed, a 30 nm pentacene layer which serves as a *p*-type semiconductor was thermally deposited at a rate of 0.2 Å/s in a vacuum evaporator at a base pressure of 3 × 10^**6**^ Torr. Finally, the Au source and drain electrodes were thermally deposited at a rate of 0.2 Å/s through a shadow mask with channel length and width of 50 μm and 1000 μm in the same evaporator at the same vacuum condition. For comparison, a control device with pentacene grown on bare SiO_2_ was fabricated simultaneously.

### Characterization

The structure of prepared Eu(tta)_3_ppta was confirmed by ^1^H NMR spectroscopy (Bruker Avance-III). The absorption spectra were collected by a LAMBDA 750 UV/Vis/NIR Spectrophotometer. The photoluminescence emission and excitation spectra of Eu(tta)_3_ppta were performed using a HORIBA FluoroMax-3 Spectrofluorometer. Atomic force microscopy (AFM, VEECO Multimode V) was used to investigate the pentacene film morphology in a tapping mode. For the light source, a commercial ultraviolet lighting emitting diode (UV-LED) was used in this study. The emitting spectrum of the UV-LED was recorded by a Hitachi FL-4600 spectrometer, and the power density was measured by a Newport Power/Energy Meter (Model 841-PE). All the electrical properties were measured using a Keithley 2612 source meter in an Mbraun nitrogen glove box. The threshold voltage (V_TH_) is calculated from the intercept of liner plot of the square root of drain current (I_DS_^½^) versus the gate voltage (V_GS_). The field-effect mobility (μ) is calculated from the saturation regime according to the equation:


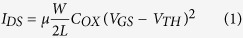


where W and L are the channel width and length, C_OX_ is the oxide dielectric (SiO_2_) capacitance per unit area, V_GS_ is the applied gate bias, V_TH_ is the threshold voltage and I_DS_ is the drain current.

## Additional Information

**How to cite this article**: Zhuang, J. *et al.* Photo-reactive charge trapping memory based on lanthanide complex. *Sci. Rep.* **5**, 14998; doi: 10.1038/srep14998 (2015).

## Supplementary Material

Supporting information
