# Supplementary material for: Photo-reactive charge trapping memory based on lanthanide complex
Source: Sci Rep. 2015 Oct 9;5:14998. doi: 10.1038/srep14998 (PMC4598868; doi:10.1038/srep14998)
Supplement: Supporting information [file srep14998-s1.doc]

Supporting Information

**Photo-reactive charge trapping memory based on lanthanide complex**

Jiaqing Zhuang^1^, Wai-Sum Lo^2^, Li Zhou^1^, Qi-Jun Sun^1^, Chi-Fai Chan^3^, Ye Zhou^1^, Su-Ting Han^1^, Yan Yan^1^, Wing-Tak Wong^*2^, Ka-Leung Wong^3^, and V. A. L. Roy^*1,4^

^1^Department of Physics and Materials Science and Center of Super-Diamond and Advanced Films (COSDAF), City University of Hong Kong, Hong Kong SAR,

^2^Department of Applied Biological and Chemical Technology, The Hong Kong Polytechnic University, Hung Hum, Hong Kong SAR,

^3^Department of Chemistry, Hong Kong Baptist University, Kowloon Tong, Hong Kong SAR,

^4^Shenzhen Research Institute, City University of Hong Kong, High-Tech Zone, Nanshan District, Shenzhen, 518057, China.

*E-mail: [val.roy@cityu.edu.hk](mailto:val.roy@cityu.edu.hk) & [wing-tak.wong@polyu.edu.hk](mailto:wing-tak.wong@polyu.edu.hk)

**Figure S1**. ^1^H NMR spectrum of Eu(tta)_3_ppta.


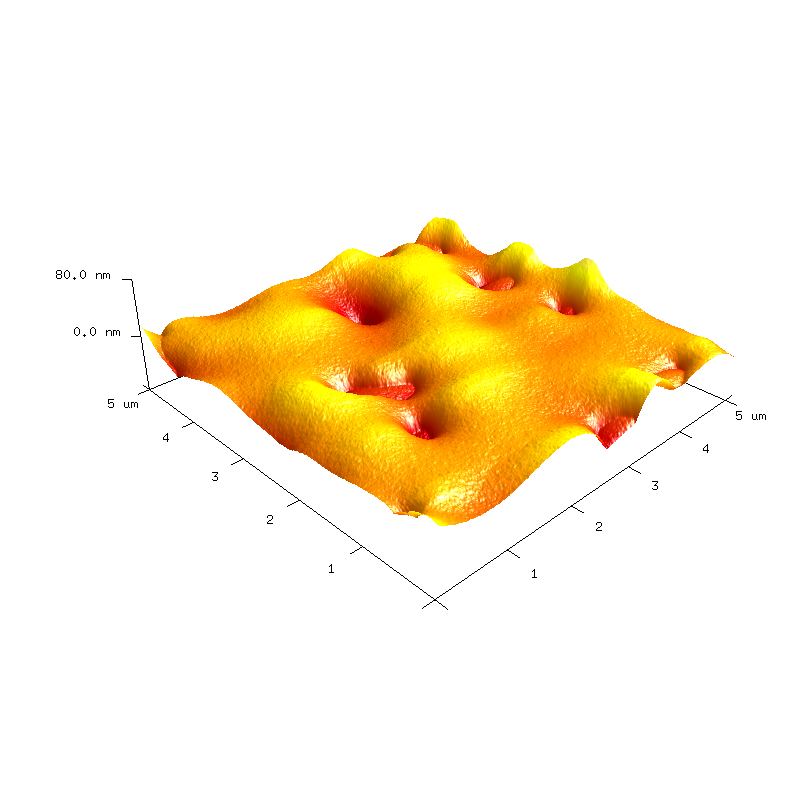


**Figure S2**. AFM image of Eu(tta)_3_ppta film.


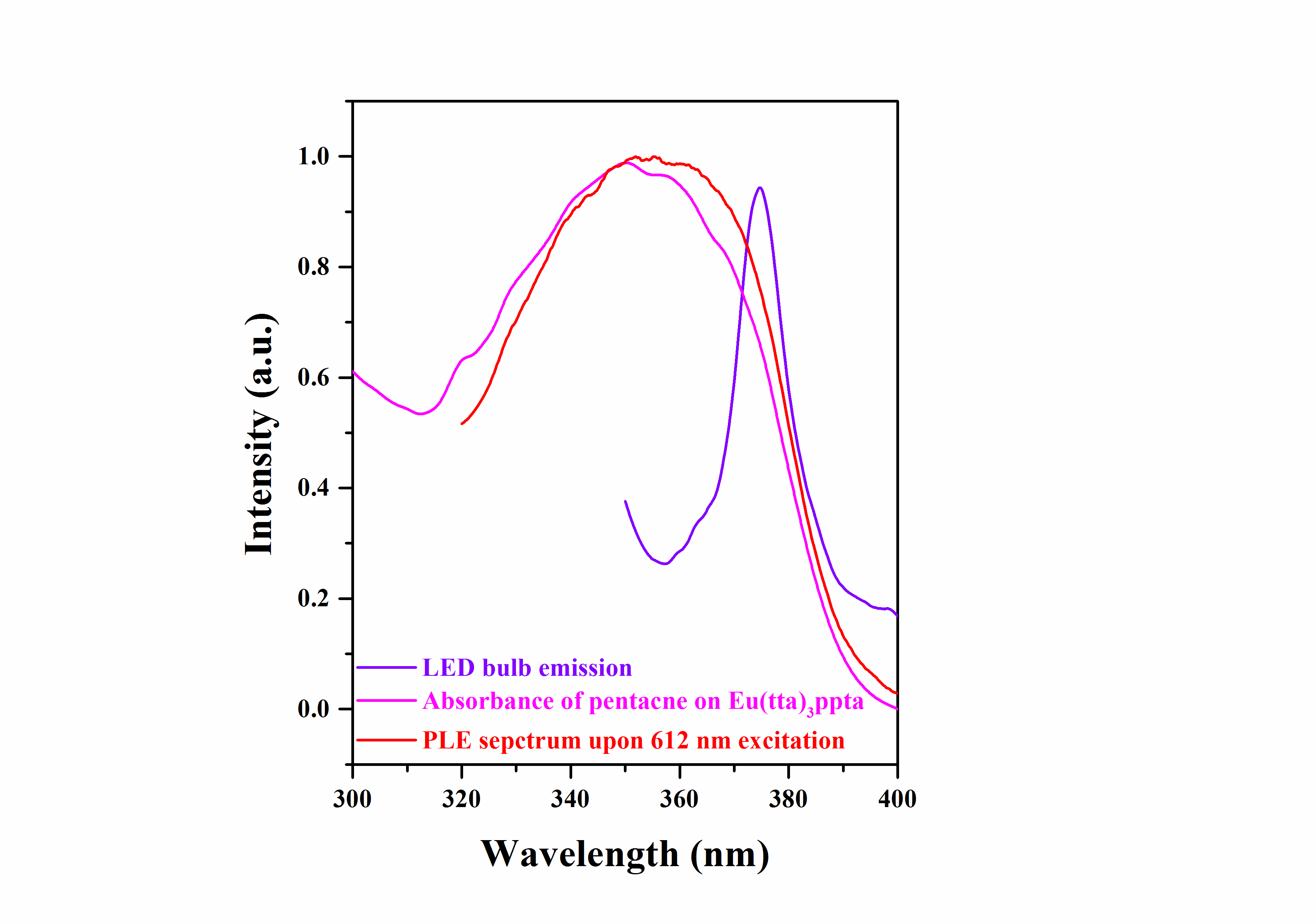


**Figure S3.** The overlap of UV LED bulb emission spectrum and absorbance of pentacene on Eu(tta)_3_ppta film.


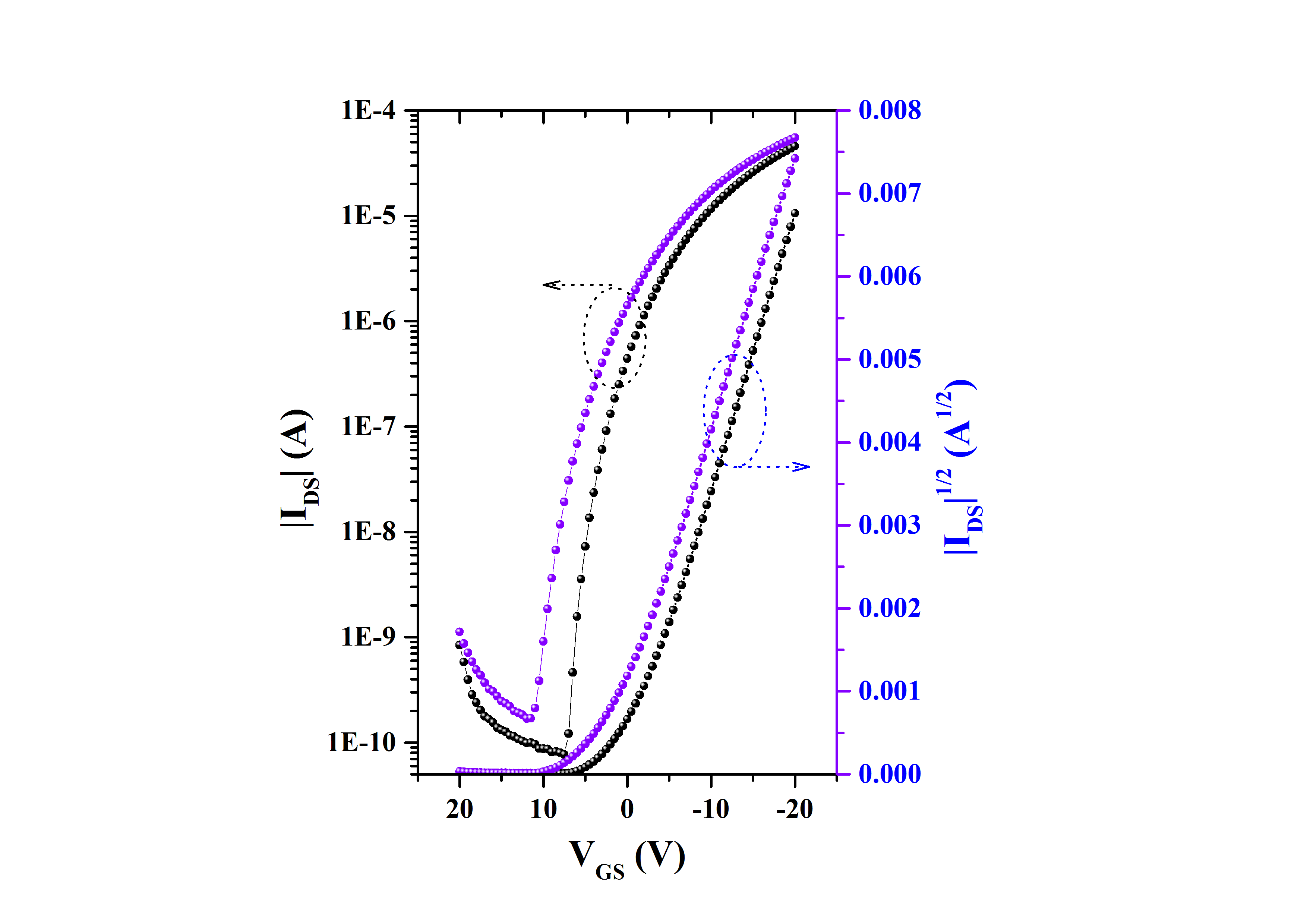


**Figure S4**. Transfer characteristic of control device with pentacene grown on bare SiO_2_ measured in dark and under UV LED bulb irradiation.


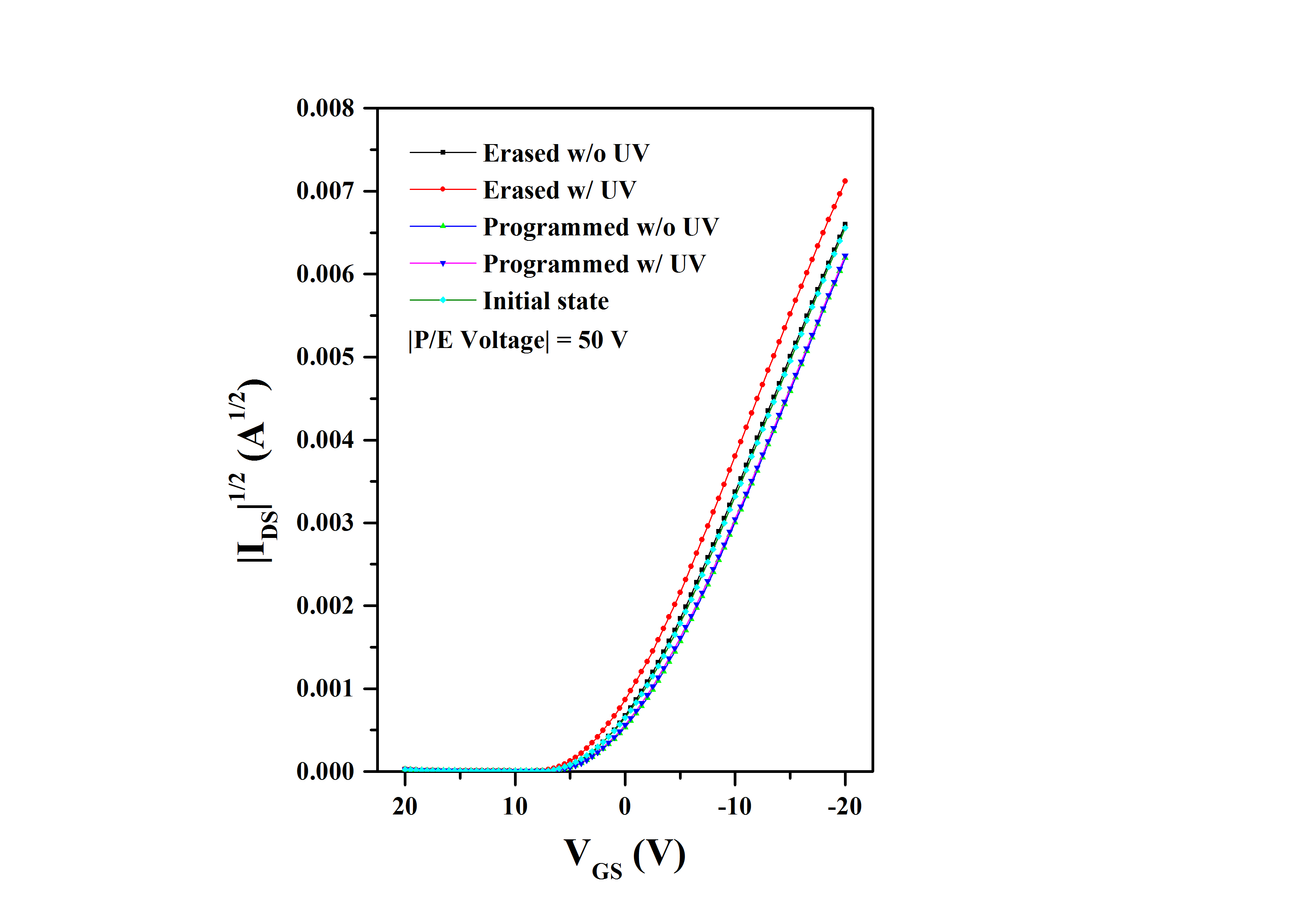


**Figure S5**. Transfer characteristics of control device with bare SiO_2_ at initial, programmed, erased states after which operated with and without assistance of UV light.
